# Thermal expansion coefficient determination of pure, doped, and co-doped anatase nanoparticles heated in sealed quartz capillaries using *in-situ* high-temperature synchrotron radiation diffraction

**DOI:** 10.1016/j.heliyon.2020.e04501

**Published:** 2020-07-30

**Authors:** Hani Manssor Albetran

**Affiliations:** Department of Basic Sciences, College of Education, Imam Abdulrahman Bin Faisal University, Dammam 31451, Saudi Arabia

**Keywords:** Materials Science, Nanotechnology, Thermal expansion coefficient, Anatase, Titania, Nanoparticles, Synchrotron radiation diffraction

## Abstract

Synchrotron radiation diffraction was conducted *in-situ* and at high temperature to establish the lattice parameters of pure/undoped, doped, and co-doped anatase nanoparticles. The nanoparticles were heated from room temperature to 950 °C in sealed quartz capillaries. The effect of pressure, doping (aluminium or indium), and co-doping (indium-chromium or silver-chromium) on the thermal expansion coefficients of nanocrystalline anatase was established. Synchrotron radiation diffraction *at* high temperature *and in-situ*, transmission electron microscopy, and the Rietveld refinement method were used to characterise the anatase nanoparticles. The anisotropy of the thermal expansion, α_a_/α_c_, for pressurised anatase nanoparticles was smaller than that for anatase heated in unpressurised air or argon, and it was much smaller in a vacuum.

## 1 Introduction

Titania (TiO_2_) or titanium (IV) dioxide is an important industrial compound that is used in catalysis, dielectrics, and pigments. It affects the local aqueous geochemistry and is an important component of soils. Ultrafine crystalline nanostructured titanium oxide has a high surface area with unusual electrical, optical, and catalytic properties and therefore, has attracted extensive interest [1, 2, 3, 4, 5, 6, 7].

Crystalline modifications of titanium dioxide include anatase, rutile, and brookite. Anatase is a common TiO_2_ crystal phase with a tetragonal crystal structure; a space group of I_41_/amd; atoms that share four edges per cell (Z); and lattice parameters (at room temperature) of *a* = 0.3784 nm, *b* = 0.3784 nm, and *c* = 0.9515 nm. The four Ti–O distances are 0.1937 (3) nm and the two Ti–O distances are 0.1964 (9) nm. With an increase in temperature, the strain energy of the lattice increases because of dislocation of TiO_6_ octahedra via their connection at four centres. The resultant lattice transformation is to rutile [8].

The wide-bandgap anatase (~3.2 eV) features make it more useful than narrow bandgap semiconductors and allows it to function at a higher temperature at which bandgaps tend to shrink [9]. Thus, anatase can be applied in high-temperature and power switching applications and in solid-state lighting to reduce the quantity of energy needed. Anatase optical absorption can be improved and changed by doping or two-atom co-doping into anatase [10, 11]. Moreover, solar-cell thermal expansion changes the semiconductor band gap [12] and affects the electron transport and cell efficiency. The parameters of solar cells with titania thin films, such as their structural and electronic and optical properties, are affected by thermal expansion [13, 14, 15, 16]. Macroscopic anatase crystal high-precision unit-cell parameters can be used as a baseline reference to study the structural changes that occur when particles expand and growth thermally. High-quality unit cell parameters of anatase and rutile were obtained at room temperature by powder camera measurements for the first time by Cromer and Herrington [17]. Rao and his co-workers calculated anatase and rutile thermal expansion for 300–1000 K by using a similar technique [18]. X-ray diffraction studies have been used to refine the unit cell parameters of anatase and rutile according to temperature [19, 20, 21, 22, 23].

The transformation of anatase-to-rutile has been studied for 400–1200 °C [24, 25, 26, 27, 28, 29, 30, 31, 32, 33, 34, 35, 36], but limited titania thermal-expansion data exist near the transition temperature. Titania thermal expansion has not been studied in detail and the effects of form, crystal structure, synthesis, doping, particles size, surface area, and atmospheric reactions from a high to a transition temperature are unknown. Pressure and doping effects on anatase nanopowder thermal expansion coefficients (TECs) with a change in temperature and atmosphere are especially unclear.

**Figure 1:**
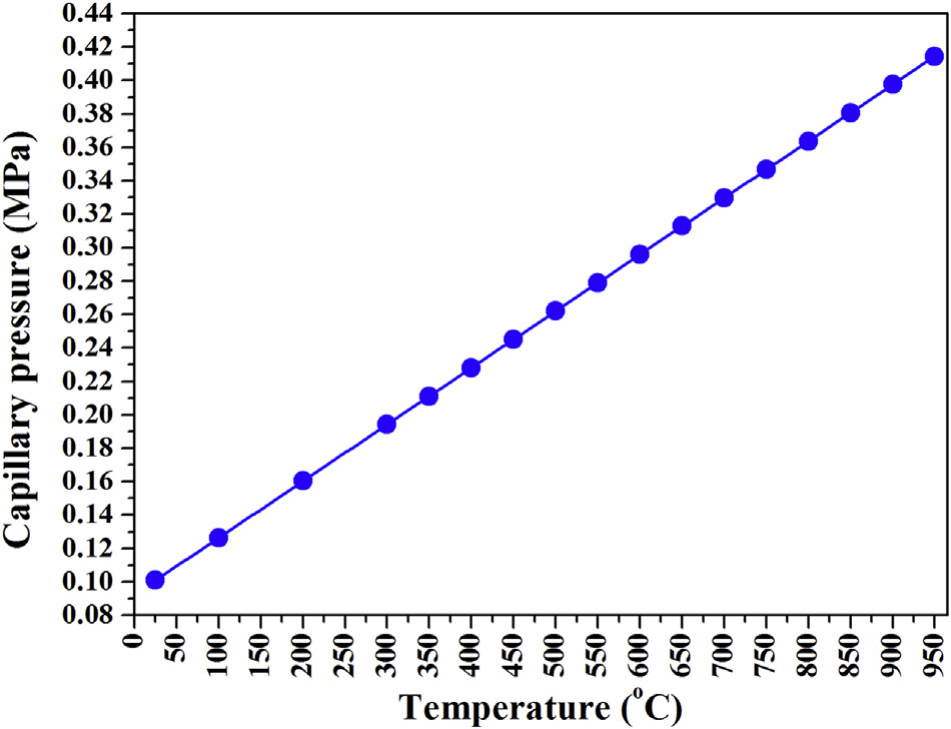
Gay-Lussac's law: capillary pressure versus temperature.

The TECs of pure, doped, and co-doped anatase nanoparticles in sealed capillaries were reported. The Rietveld refinement method was used to analyse synchrotron diffraction patterns at various temperatures and the anatase lattice parameters in sealed capillaries are discussed for 25–950 °C. Gay–Lussac's Law for heating in a sealed capillary was used to study changes for 0.10–0.42 MPa and 25–950 °C, respectively (Figure 1).

## 2 Experimental methods

### 2.1 Material synthesis

The effect of doping and co-doping on anatase TEC was studied based on pure, In-doped, Al-doped, co-doped Ag–Cr, and co-doped In–Cr titania nanoparticles. Information on pure/undoped anatase nanopowder and that doped with In and (In–Cr) has been provided previously [33, 35, 36]. A sol–gel approach was taken using titanium (IV) isopropoxide (C_12_H_28_O_4_Ti, molecular weight of 284.22 g/mol, Sigma-Aldrich, New South Wales, Australia). Samples were synthesised with magnetic stirring at 100 °C for 60 min to achieve organic material evaporation before room-temperature drying for 48 h. Titania precursor titanium (IV) isopropoxide and nanohydrate indium (III) nitrate (InN_3_O_9_.xH_2_O, molecular weight 300.83 g/mol), aluminium nitrate nonahydrate (AlN3O_9_.9H_2_O, molecular weight 375.13 g/mol), silver nitrate solution (AgNO_3_, molecular weight 169.87 g/mol), and nanohydrate chromium (III) nitrate (CrN_3_O_9_.9H_2_O, molecular weight 400.15 g/mol) from Sigma-Aldrich were used to synthesize In-doped, Al-doped (both 1 wt%, calculated), Ag–Cr co-doped, and In–Cr co-doped (0.5 wt% of each element, calculated) titania nanoparticles by the same method. The doping concentrations were increased to ~4 wt% and ~2 wt% for the In- and Al-doped and Ag–Cr and In–Cr co-doped samples, respectively by evaporation of the organic material by magnetic mixing at 100 °C for 1 h. Sample (2.4 g) was produced by room-temperature drying for 48 h.

### 2.2 Morphological characterization

Samples (~5 mg) were ground and ultrasonicated in ethanol for 5 min and studied by transmission electron microscopy (TEM) after synchrotron radiation diffraction (SRD) at high temperature under *in-situ* conditions (see Section 2.3). Imaging of two droplets of suspension on carbon-coated copper grids was achieved by using an EFI Morgagni 268 TEM (LaB6 filament, 100 kV, Institute for Research & Medical Consultations, Imam Abdulrahman Bin Faisal University, Saudi Arabia).

### 2.3 SRD at high temperature under in-situ conditions

The high-temperature SRD approach *in-situ* has been described previously [33, 35, 36]. Ground samples were loaded to one third into sealed and funnel-shaped quartz capillaries with open ends and with an 80 mm length and 1.00 mm diameter in an ultrasonic bath (Charles Supper Company, USA). A nitrogen flame was used to seal the broken capillary's open funnel side. The capillary was inserted into 10-mm brass stub holders for SRD analysis at the Australian Synchrotron (Mythen II microstrip X-ray detector; acquisition time of 3 min per SRD pattern, room temperature, 100 °C increments at 2 °C/min from 100 °C to 300 °C, 50 °C increments from 350 °C to elevated temperature; 15.0204 keV photon energy, λ = 0.0825 nm, and 2θ = 5–84°. The Debye–Scherrer geometry was used to obtain the SRD patterns.

**Figure 2:**
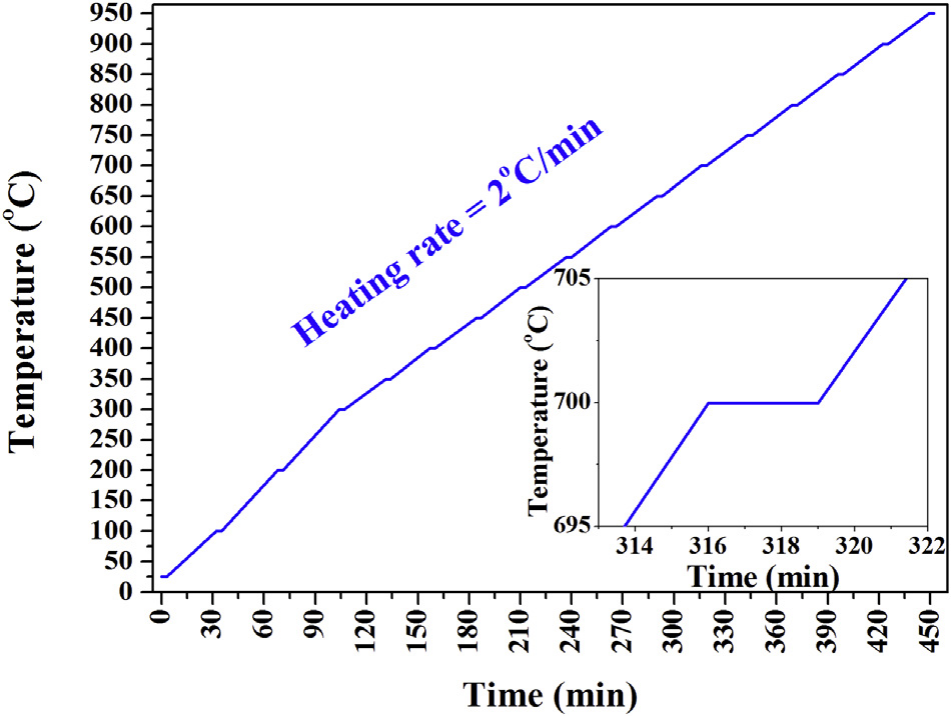
Non-isothermal SRD heating protocol (acquisition of SRD data in 3-min).

The SRD heating protocol contained plateaus in the non-isothermal SRD heating *in-situ* (Figure 2) and represents the 3-min SRD data-acquisition periods. Pure anatase nanoparticles, Al-doped and Ag–Cr co-doped anatase nanoparticles, and anatase nanoparticles that were In-doped and co-doped with In–Cr were studied to 800 °C, 900 °C, and 950 °C, respectively. The upper 950 °C limit was chosen to determine the effect of temperature on particle size and because there was a limited transformation of pure anatase to rutile below 800 °C [33, 36].

### 2.4 Analysis of anatase lattice parameters

DIFFRAC. EVA 3.1 software (International Centre for Diffraction Data powder-diffraction file database) was used for phase identification through peak position and intensity matching. TOPAS software (Bruker AXS, Version 4.2) was used to fit the anatase crystal structures (ICSD 202242) and the anatase lattice parameters. The temperature-dependent lattice parameters were obtained from Rietveld refinements, and the *2θ*_*0*_, parameters of the peak shape, background of the pattern, and scale factor were obtained over a range of temperatures.

## 3 Results and discussion

### 3.1 Microstructure imaging by TEM

**Figure 3:**
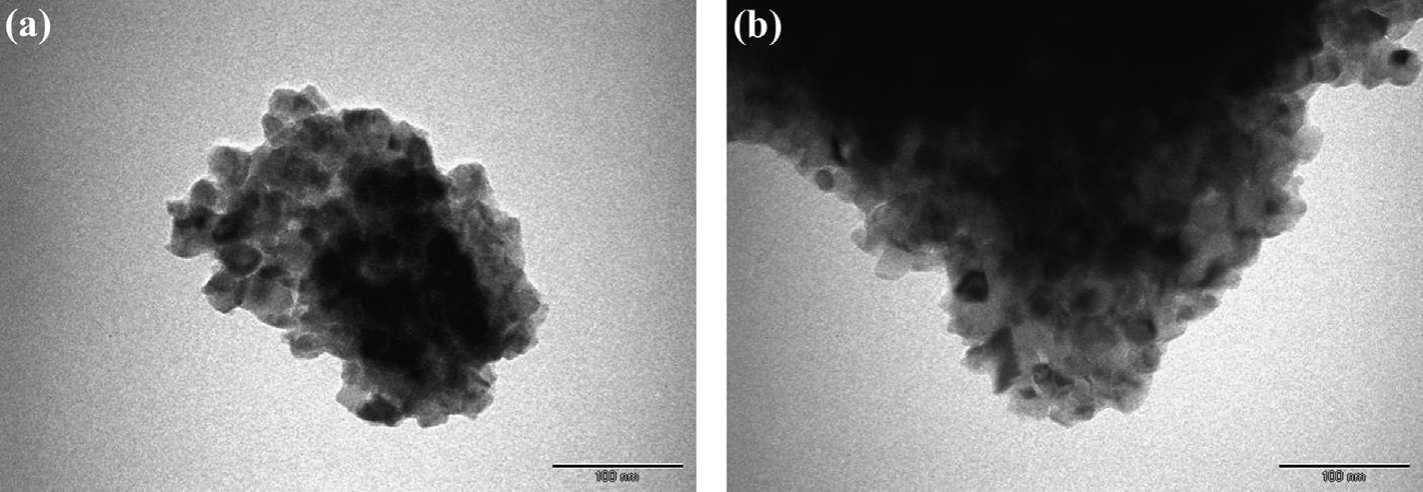
TEM micrographs: pressurised (a) Al-doped, and (b) Ag–Cr co-doped anatase nanoparticles.

The nanopowder morphology and microstructure was studied by TEM. Characteristic TEM images for Al and Ag–Cr co-doped titania nanoparticles after heating to 900 °C are shown in Figure 3(a), (b), respectively. Secondary electron images of nanoparticles of pure anatase that had been heated to 800 °C, and In-doped, and titania nanoparticles that were co-doped with In–Cr and heated to 950 °C have been provided elsewhere [33, 36]. The uniform pure/undoped anatase nanopowder [33] size increased from 85 ± 31 nm to 178 ± 44 nm for In-doped titania, and to ~218 ± 64 nm for In–Cr co-doped titania nanopowder particles heated to 950 °C (Table 1). They formed non-homogeneous, variable, nonspherical particles with a strict shape tolerance. Crystallite agglomerations ~34 ± 7 nm formed titania nanoparticles that were ~200 nm Al-doped and co-doped with Ag–Cr (consistent with preliminary doped and co-doped titania nanoparticles [33, 36]), which indicates that an increased temperature promoted grain growth and titania phase transformation.

**Table 1:** Electron micrograph particle sizes (nm) of pressurized titania nanoparticles after calcining and cooling to room temperature.

Pressurized titania	Particles sizes (nm)	Temperature (°C)	Reference
Pure	85 ± 31	800	[33]
Al-doping	232 ± 47	900	This study
In-doping	178 ± 44	950	[36]
In–Cr co-doping	218 ± 64	950	[36]
Ag–Cr co-doping	221 ± 32	900	This study

### 3.2 SRD patterns from sealed capillary

**Figure 4:**
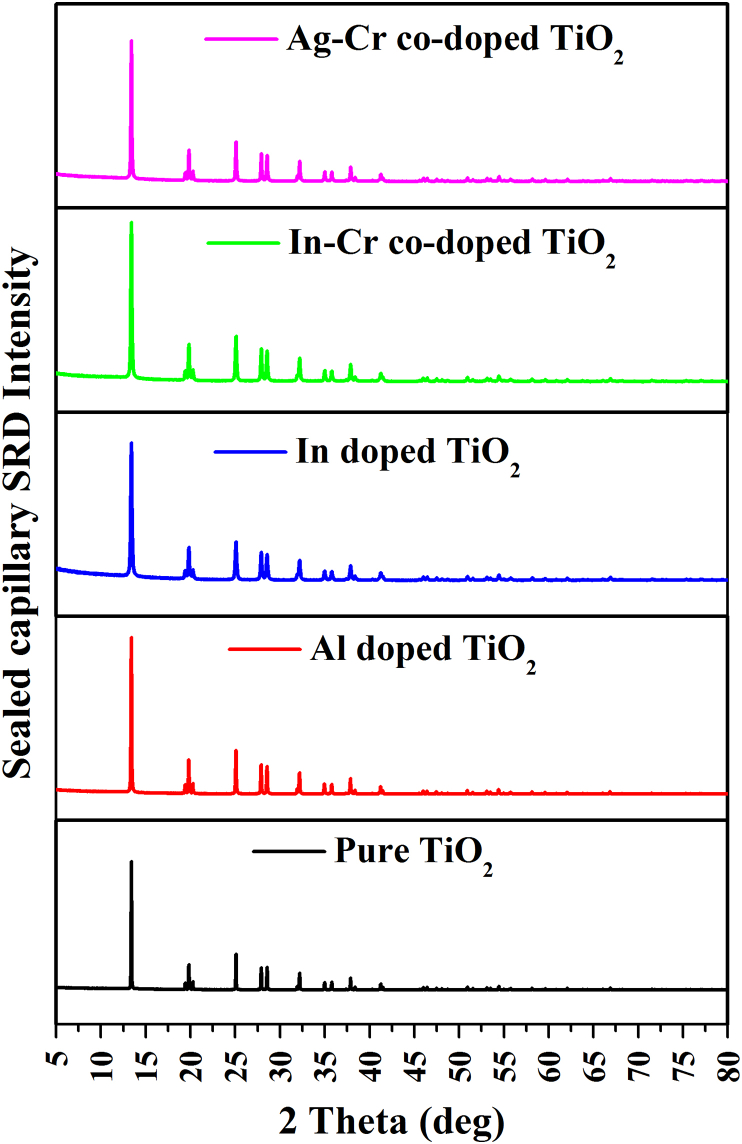
SRD plots for pure anatase nanoparticles and those doped with Al, In, In–Cr, and Ag–Cr. The anatase nanoparticles were heated at 750 °C and ~0.34672 MPa in sealed capillaries.

**Figure 5:**
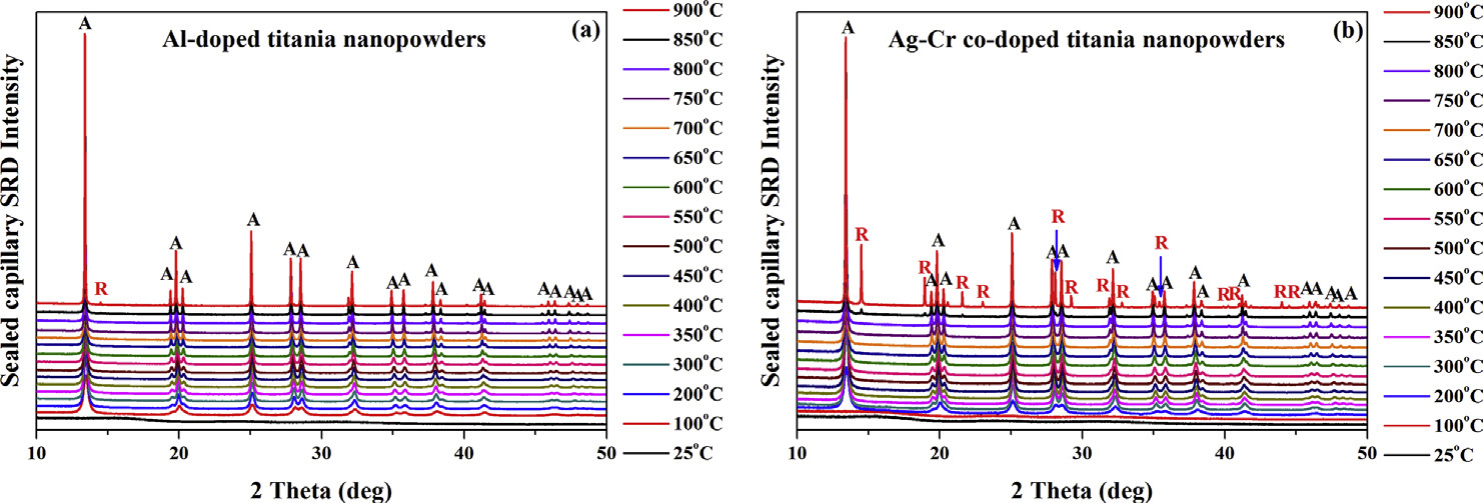
High-temperature SRD plots under *in-situ* conditions for titania nanoparticles that were heated from 25 to 900 °C in sealed capillaries. The nanoparticles were doped with (a) Al, and (b) Ag–Cr [A and R represent anatase and rutile, respectively].

Figure 4 shows typical stacked SRD plots at 750 °C (~0.34672 MPa) for the nanoparticles. Al-doped and co-doped Ag–Cr titania nanopowder stacked SRD plots from 25 to 900 °C are shown in Figure 5(a), (b), respectively. At 200 °C, crystalline anatase formed for Ag–Cr co-doping, which contrasts with a crystallization temperature of pressurised titania for pure anatase nanoparticles and those doped with In and In–Cr reported in the literature [33, 36]. The rate of amorphous-to-anatase transformation is accelerated by heating under pressure whereby anatase formed in an environment that was oxygen rich, at lower temperature, and with an increased partial pressure in the capillaries that were sealed at ~0.16 MPa and 200 °C (Figure 1). Such an observation has been made for an oxygen working pressure and transformation on a TiO_2_ film on a substrate of glass with a pulsed laser [37]. Therefore, anatase formation at a lower temperature results from an oxygen-rich environment inside the sealed capillary that results from an increase in oxygen partial pressure with increasing temperature and propanol desorption with oxygen (27%). A linear increase in gas pressure inside the sealed capillary from ~0.10 MPa at 25 °C to ~0.39756 MPa at 900 °C prevented the anatase-to-rutile transformation. A higher temperature and gas pressure resulted because of interstitial Ti formation and an improved structural rigidity, which prevented the relaxation of Ti bonds that is required for the anatase-to-rutile transformation. Elemental aluminium in the Al-doped anatase nanoparticles decreased the anatase crystallization temperature to 100 °C. A partial replacement of 0.061-nm-radius Ti ions by 0.0535-nm-radius Al ions facilitates the transformation of amorphous phase to anatase in the Al-doped material [29]. Cr and V ions show a similar effect [25, 28]. As an alternative to interstitial implantation at normal pressure, these ions substitute into the Ti sub-lattice. These interstitial ions inhibit titania transformation, whereas substitutional ions can either inhibit or accelerate phase transformation [38, 39]. At elevated temperature, the higher gas pressure yielded interstitial ions of Ti and possibly interstitial Al formation, and enhanced the structural rigidity more than the smaller Al ions in Al-doping. In the latter case, essential Ti bonding relaxation that is required for anatase-to-rutile transition at elevated temperature and pressure (800 °C, 0.36367 MPa) did not occur.

### 3.3 TECs

**Figure 6:**
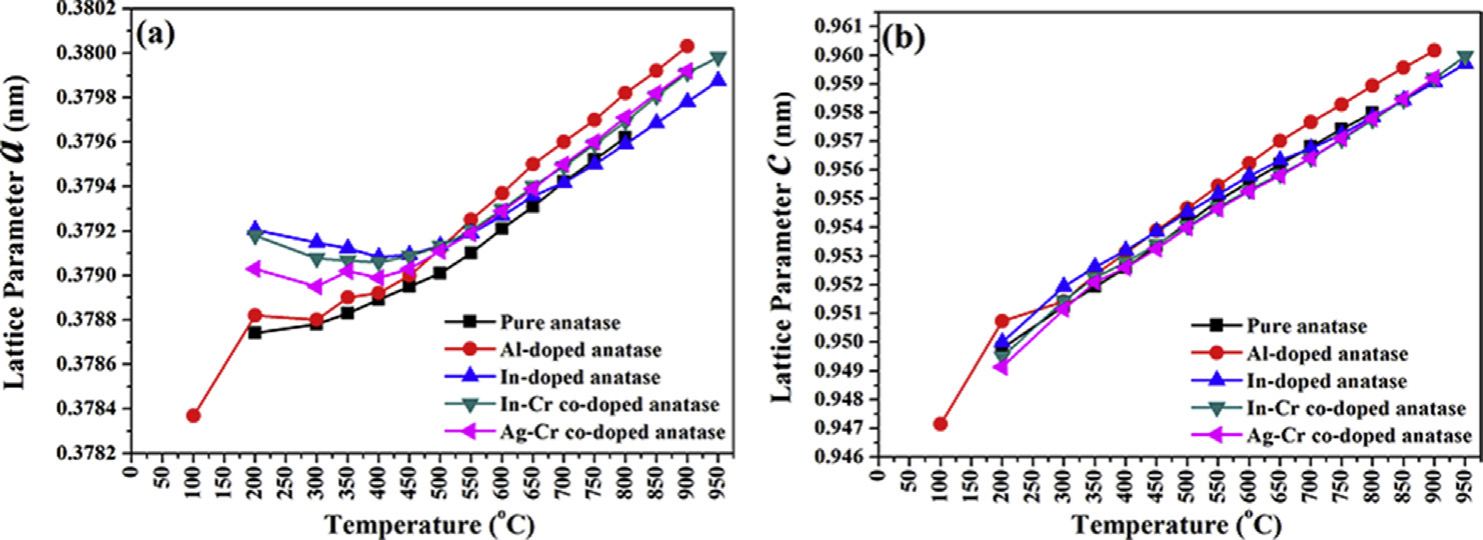
Anatase lattice parameters (a) *a* and (b) *c* as a function of temperature for pure/undoped, In-doped, Al-doped, In–Cr co-doped, and Ag–Cr co-doped anatase nanoparticles.

**Figure 7:**
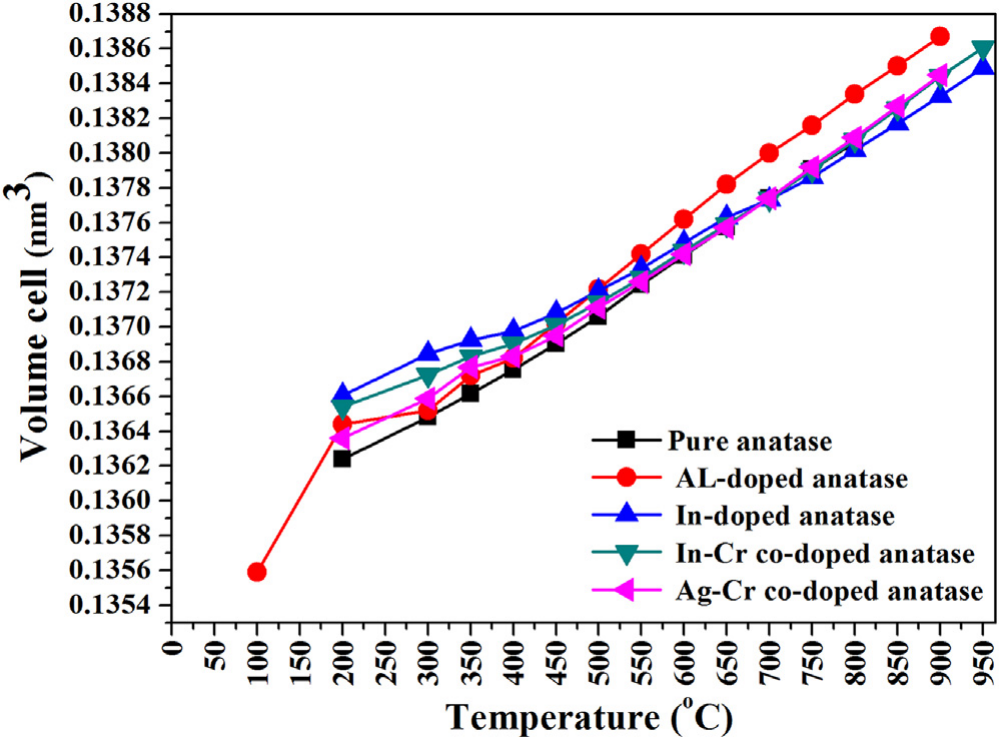
Cell volume variation with temperature for anatase in pure/undoped, In-doped, Al-doped, In–Cr co-doped, and Ag–Cr co-doped anatase nanoparticles.

**Figure 8:**
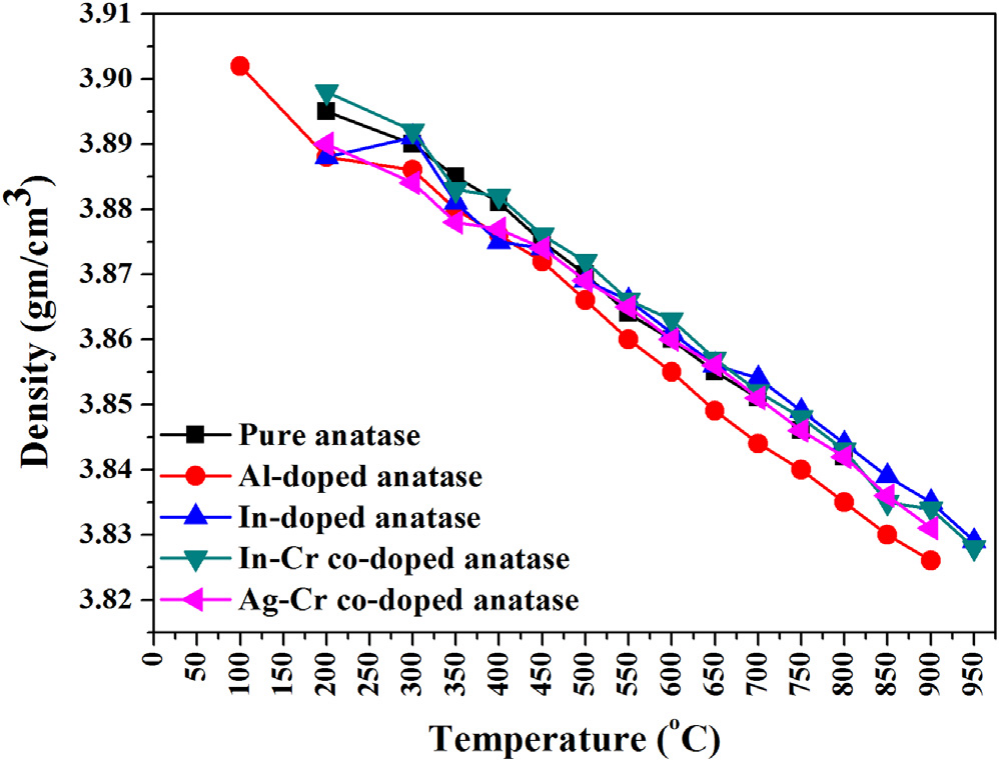
Variation of anatase density with temperature in pure, In, In–Cr, Al, and Ag–Cr doped anatase nanoparticles.

**Figure 9:**
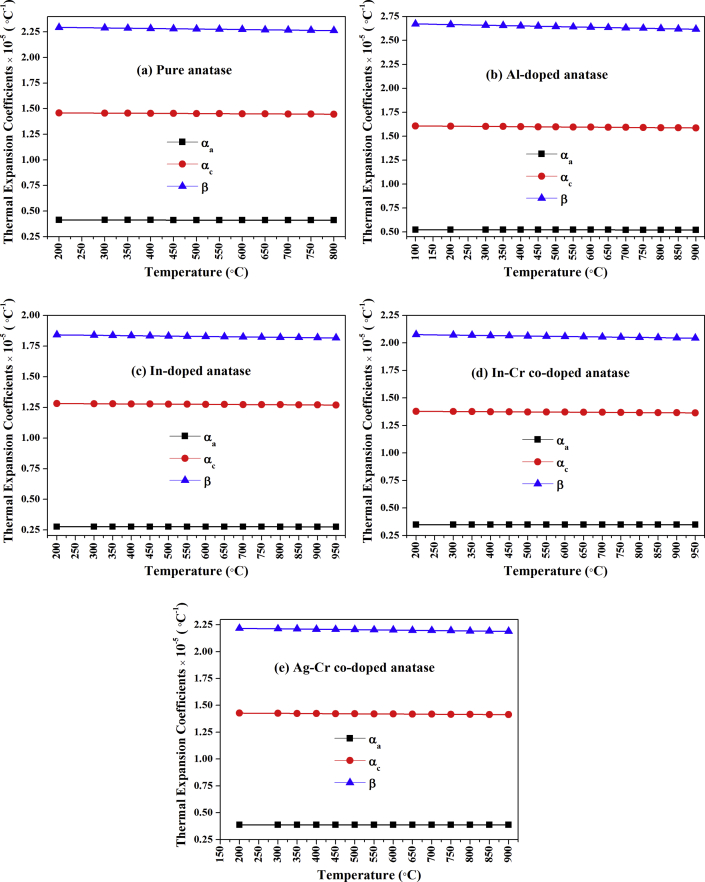
Variation of TECs with temperature for (a) pure anatase nanoparticles and nanoparticles that were doped with (b) Al, (c) In, (d) In–Cr, and (e) Ag–Cr.

Figures 6(a), (b) and 7 show unit cell parameters and cell-volume variations for the anatase nanoparticles at 25–950 °C. The anatase unit cell parameter grew-nonmonotonically, and the anatase density decreased (Figure 8) with an increase in temperature. Calculated and literature linear (α_a_, α_c_) and volumetric (*β*) TECs at 750 °C (from Figures 6 and 7) are shown in Table 2 [18,23,27,40]. The average linear (α_a_, α_c_) and volumetric (*β*) TECs were determined from the slope of the expansion curve to 800 °C, 900 °C, and 950 °C from room temperature for pure and doped anatase nanoparticles (Al, In, Ag–Cr, In–Cr). Changes in anatase lattice parameters and linear (α_a_, α_c_) and volumetric (*β*) anatase TECs will be used to incorporate the influence of high pressure, doping, and co-doping in the anatase structure. TECs of anatase heated in air and vacuum appear to be influenced by an increase in oxygen partial pressure. A comparison of TECs for α_*a*_ and α_*c*_ and the volume expansion coefficient of anatase given by *β =2α*_*a*_ *+ α*_*c*_, and the anisotropy of the thermal expansion, α_a_/α_c_ for pure, Al-doped, In-doped, In–Cr co-doped, and Ag–Cr co-doped anatase nanoparticles is provided in Table 2. The vibration amplitude increases the TEC, and so *α*_*a*_ is lower than the reported powdered TiO_2_ that was heated at ambient pressure in air [18, 40], and much lower than for powdered material heated in a vacuum [23]. A lower anatase linear expansion coefficient ratio of *α*_*a*_ to *α*_*c*_ resulted than for anatase heated in air and/or vacuum. The vibration amplitude decreased as a result of the lower anatase TECs for the sealed capillary, which resulted from the high oxygen pressure, and the anatase atom thermal expansion at elevated temperature decreased as a result. The variation of thermal expansion coefficients with temperature is shown in Figure 9. An increase in temperature resulted in a decrease in anatase coefficient with an increase in temperature because the gas pressure increased with temperature. The anatase coefficient temperature dependence is given by:

(a) Pure anatase:

(1) $$\alpha_{a}=-1.69637\times \;10^{-11}\;T+0.412761\;\times \;10^{-5}$$

(2) $$\alpha_{c}=-2.10871\times \;10^{-10}\;T+1.46289\;\times \;10^{-5}$$

(3) $$\beta =-5.18632\times \;10^{-10}\;T+2.30394\;\times \;10^{-5}$$

(b) Al-doped anatase:

(4) $$\alpha_{a}=-2.71965\times \;10^{-11}\;T+0.522839\;\times \;10^{-5}$$

(5) $$\alpha_{c}=-2.54726\times \;10^{-10}\;T+1.60917\;\times \;10^{-5}$$

(6) $$\beta =-6.98862\times \;10^{-10}\;T+2.67965\;\;\times \;10^{-5}$$

(c) In-doped anatase:

(7) $$\alpha_{a}=-0.763996\times \;10^{-11}\;T+0.276788\;\times \;10^{-5}$$

(8) $$\alpha_{c}=-1.62608\times \;10^{-10}\;T+1.28442\;\times \;10^{-5}$$

(9) $$\beta =-3.34191\times \;10^{-10}\;T+\;1.84766\;\;\;\times \;10^{-5}$$

(d) In–Cr co-doped anatase:

(10) $$\alpha_{a}=-1.21196\times 10^{-11}\;T+0.348804\;\;\;\times \;10^{-5}$$

(11) $$\alpha_{c}=-1.88033\times \;10^{-10}\;T+1.38209\times \;10^{-5}$$

(12) $$\beta =-4.2377\times \;10^{-10}\;T+2.08334\times \;10^{-5}$$

(e) Ag–Cr co-doped anatase:

(13) $$\alpha_{a}=-1.50309\times \;10^{-11}\;T+0.388502\;\;\times \;10^{-5}$$

(14) $$\alpha_{c}=-2.01769\times \;10^{-10}\;T+1.43163\;\times \;10^{-5}$$

(15) $$\beta =-4.03844\times \;10^{-10}\;T+2.22514\times \;10^{-5}$$

where *T* is the temperature in °C.

**Table 2:** Anatase TECs calculated from Figures 6 and 7 at 750 °C, and from literature: linear (*α*_*a*_, *α*_*c*_) and volumetric (*β*).

TEC ×10^−5^ (°C^−1^)	(This study) Pressurised					Hummer *et al.* [40]	Albetran *et al.* [27]		Rao *et al.* [18]	Jagtap *et al.* [23]	
Environment	Pure	Al	In	In–Cr	Ag–Cr	Air	Air	Argon	Air	Air	Vacuum
*α*_*a*_	0.41 (3)	0.52 (2)	0.28 (4)	0.35 (4)	0.39 (4)	0.58 (1)	0.72 (15)	0.62 (49)	0.63 (3)	0.63 (1)	1.20 (1)
*α*_*c*_	1.45 (2)	1.59 (5)	1.27 (3)	1.37 (2)	1.42 (3)	1.03 (2)	0.86 (84)	0.79 (50)	1.29 (6)	0.84 (2)	1.18 (2)
*β*	2.27 (4)	2.62 (8)	1.82 (5)	2.05 (7)	2.19 (6)	2.17 (4)	2.29 (11)	2.00 (48)	2.54 (11)	2.13 (4)	3.59 (4)
*α*_*a*_/*α*_*c*_	0.28 (2)	0.32 (1)	0.22 (2)	0.26 (2)	0.28 (2)	0.56 (1)	0.84 (1)	0.75 (1)	0.49 (3)	0.75 (1)	1.02 (1)

### 3.4 Crystallite size of pressurized anatase nanoparticles

**Figure 10:**
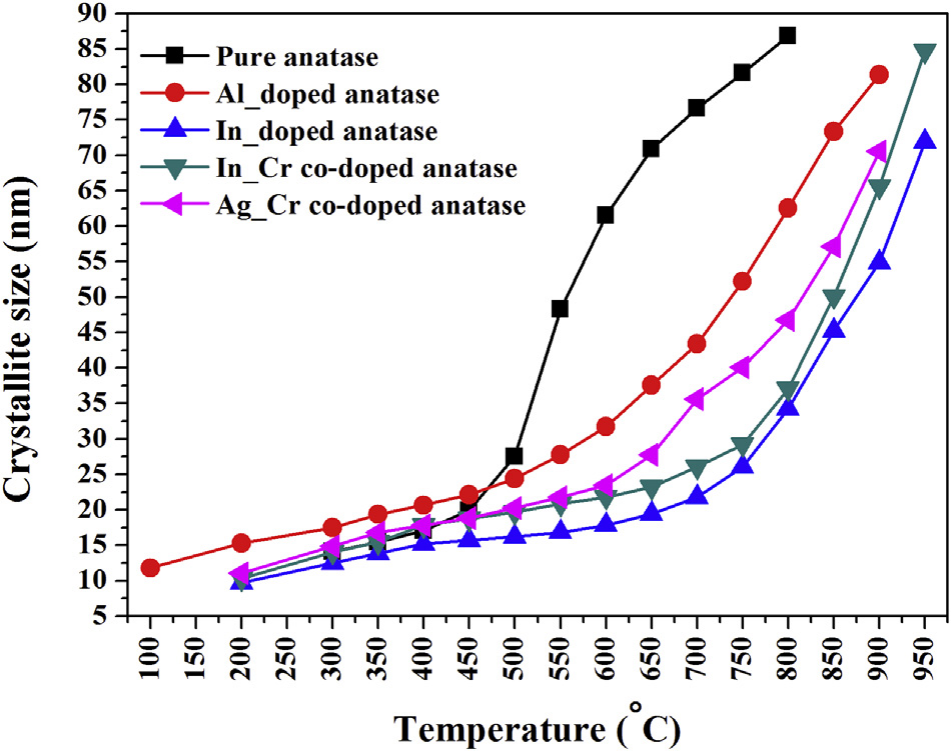
Variation of crystallite size with temperature for pure anatase nanoparticles and nanoparticles that were doped with Al, In, In–Cr, and Ag–Cr.

The Debye–Scherrer's equation was used to calculate the average crystallite size (L) for pure, Al-doped, In-doped, In–Cr co-doped, and Ag–Cr co-doped anatase nanopowder from 25 °C to 950 °C from the (101) reflection [41]:

(16) $$L=\frac{k\lambda }{\beta \;\cos\;\theta }$$

where *k*, λ, *θ*, and *β* are the shape factor (0.94), X-ray wavelength (0.0825 nm), Bragg angle, and full width at half maximum (line broadening at half of the maximum intensity) in radians, respectively. The temperature-dependent effect of doping, co-doping, and pressure on anatase crystallite growth is presented in Figure 10. The average anatase crystallite size increased from 12–15 nm at 200 °C to 87 nm, 63 nm, 34 nm, 37 nm, and 47 nm for pure, Al-doped, In-doped, In–Cr co-doped, and Ag–Cr co-doped anatase nanopowder, respectively, at 800 °C. Therefore, an increase in crystallite size yields a decreasing TECs, like the TEC of carbon-supported Pt nanoparticles [42]. The conversion of amorphous material to crystalline anatase through atomic diffusion-controlled nucleation and growth results because of anatase crystallite growth with temperature and oxygen gas pressure behaviour.

### 3.5 Effect of anatase thermal expansion on anatase-to- rutile phase transformation

The solid symmetry may change and a new phase with a smaller volume may form at higher pressures [43]. For In-, In–Cr, and Ag–Cr-doped titania nanoparticles at 850 °C, crystalline rutile with a *P4*_*2*_*/mnm* space group and a smaller volume than the crystalline anatase appeared. For pure anatase nanoparticles, at 900 °C, Al-doped nanoparticles showed no transformation from anatase to rutile up to 800 °C. The oxygen gas pressure led to a higher anatase stability in sealed capillaries for pressurized anatase compared with air or vacuum. Titanium atomic structures yield a symmetry change as a function of temperature and pressure because of titania electronic structures. The rutile TEC is higher than anatase [27]. The rutile phase experiences anisotropy in thermal expansion because a greater strain results in rutile compared anatase. The roles of atmosphere, impurity, and crystalline size have a significant effect, which may explain the lower TECs of anatase. The high oxygen partial pressure in environment that is rich in oxygen pressure decreases the TEC for anatase for amorphous-to-anatase phase transformation. An atmosphere that was rich in oxygen generated a high partial pressure, sped up the amorphous/anatase transformation, and reduced the anatase TECs. The high partial pressure prevented relaxation because of the breakage of anatase bonds and the formation of rutile bonds.

## 4 Conclusions

Doping, co-doping, and partial pressure affected the linear and volume TECs of anatase nanoparticles, and these effects were studied by SRD experiments at high temperature and *in-situ* in a sealed capillary on pure, In-doped, Al-doped, Ag–Cr co-doped, and In–Cr co-doped anatase nanoparticles. Rietveld analysis was used to determine the unit-cell parameters of anatase nanopowder. The main conclusions are:

• Small Al atoms were substituted into the crystal structure of the titania and led to crystalline anatase formation at 200 °C from pure amorphous material and titania nanoparticles that had been doped with In, In–Cr, and Ag–Cr at 100 °C for Al-doped titania nanoparticles.
• Rutile formed as crystalline titania at 850 °C and 900 °C for Al-doped nanoparticles.
• The unit cell parameter of anatase grew nonmonotonically, and its density decreased with an increase in temperature.
• The average linear (α_a_, α_c_) and volumetric (β) TEC decreased with an increased temperature and high partial pressure in capillaries with pure, doped, and co-doped anatase nanopowder. The oxygen partial pressure yielded results that were similar to those for anatase heated in air and vacuum.
• An increase in temperature led to a decrease in linear TEC.

A higher temperature led to an increase in the oxygen partial pressure inside sealed capillaries and reduced the average linear (α_a_, α_c_) and volumetric (*β*) TECs for pure, Al, In, In–Cr, and Ag–Cr co-doped anatase nanoparticles.

## Declarations

### Author contribution statement

Hani Manssor Albetran: Conceived and designed the experiments; Performed the experiments; Analyzed and interpreted the data; Contributed reagents, materials, analysis tools or data; Wrote the paper.

### Competing interest statement

The authors declare no conflict of interest.

### Additional information

No additional information is available for this paper.
